# A dataset of laymen olfactory perception for 74 mono-molecular odors

**DOI:** 10.1038/s41597-025-04644-2

**Published:** 2025-02-26

**Authors:** Antonie Louise Bierling, Alexander Croy, Tim Jesgarzewsky, Maria Rommel, Gianaurelio Cuniberti, Thomas Hummel, Ilona Croy

**Affiliations:** 1 Department of Clinical Psychology, Institute of Psychology, Friedrich-Schiller-University Jena, Jena, 07743 Germany; 2 Department of Psychotherapy and Psychosomatic Medicine, Faculty of Medicine and University Hospital Carl Gustav Carus, TUD Dresden University of Technology, Dresden, 01307 Germany; 3 Institute for Materials Science and Max Bergmann Center for Biomaterials, TU Dresden, Dresden, 01069 Germany; 4 Institute of Physical Chemistry, Friedrich-Schiller-University Jena, Jena, 07743 Germany; 5 Faculty of Medicine, TU Dresden, Smell and Taste Clinic, Dresden, 01307 Germany; 6 German Centre for Mental Health (DZPG), site Halle-Jena-Magdeburg, Halle-Jena-Magdeburg, Germany

**Keywords:** Human behaviour, Human behaviour, Olfactory system

## Abstract

The molecular structure of an odor determines whether and how it is perceived by humans. However, the principles of how odorant chemistry links to perceptual patterns remain largely unknown and are primarily studied using odor rating datasets from highly trained olfactory experts, such as perfumers. This limits our knowledge of typical odor perception and its variability over individuals. We provide a dataset featuring free descriptions, evaluative ratings, and qualitative labels for 74 chemically diverse mono-molecular odorants, rated by a large sample of young adults. A total of 1,227 participants described and rated the odors, and completed questionnaires covering their demographic background, personality traits, and the role of olfaction in their daily lives. The dataset offers a valuable foundation for research aimed at understanding the fundamentals of olfactory perception.

## Background & Summary

For decades, scientists have been trying to answer the question of why an odor smells the way it does^1^. Since then, research has led to the decoding of the olfactory receptor genome and outlined that only approximately 400 human olfactory receptor genes are responsible for the recognition of more than one million different olfactory percepts through olfactory pattern recognition at the level of the olfactory bulb^2^. However, the question of how exactly chemical structure relates to smell remains unsolved. Some associations have been discovered. For example, a few specific chemical groups are associated with characteristic odors, e.g., esters typically smell fruity and floral^3^, and larger and more complex molecules tend to smell more pleasant than smaller and less complex ones^4,5^. However, only very few of such general rules exist. Odors posit, besides size and complexity, hundreds or even thousands of molecular properties and all can be relevant to olfactory perception.

Unraveling the structure-percept relation hence calls for large datasets in which many participants evaluate many odors. Generating datasets of odor perception is quite time-consuming. Olfactory stimuli must be carefully prepared and stored to maintain odor quality, the presentation is only possible in an analogue fashion (compared to images or sound, which can easily be distributed digitally) and odor adaptation sets in fast with cross-effects between different odors^6^, limiting the number of odor presentation per trial. This makes large-scale approaches challenging. As a result, almost all available databases originate from trained expert panels or the perfume industry, such as those from Dravnieks^7^, Leffingwell^8^, or The Good Scent Company (dataset available from https://github.com/pyrfume, where a well-curated collection of currently available databases is stored). While such expert databases are a valuable resource for evaluations of olfactory quality and convince by high reliability, they are somewhat limited in their validity and objectivity. Most of those use predefined labels (such as “pleasant”, “sweet”, “bitter”, “musky”) which prime the smellers’ perception. The labels vary between databases, which makes comparisons challenging. Furthermore, for many of these databases, the data generation process lacks transparency, particularly concerning how perceptual labels were chosen, the number of expert or non-expert individuals involved in the evaluations, and how the evaluations of multiple experts were aggregated. Finally, ratings which are obtained from a small sample of trained experts, are not necessarily representative of the general population’s (laypeople) perception^9^. One important dataset investigating laypeople was published in 2016 by Keller & Vosshall^10^, in whose study over 450 substances were rated in several odor concentrations by 55 healthy laypeople. With their dataset, some of the previous findings in experts were replicated, e.g., a positive association between pleasantness and molecular size^10^. However, the study also revealed substantial variance in olfactory perception. This is not surprising - the perception of smells is dependent on numerous factors, from person-related factors like gender^11^, age^12,13^, and personality^13,14^, or cultural background^15,16^, to context and experience related influences on smelling^17,18^. Thus, in order to quantify and conduct more fine-tuned analyses on inter-individual variation in olfactory perception, larger datasets are necessary, ideally, with a magnitude of 10 or 20 times the number of participants as studied in Keller & Vosshall. Such data would enable a more precise investigation of the relationship between chemical structure and perception, offering a significantly improved signal-to-noise ratio by better control over person-related variability.

**Fig. 1:**
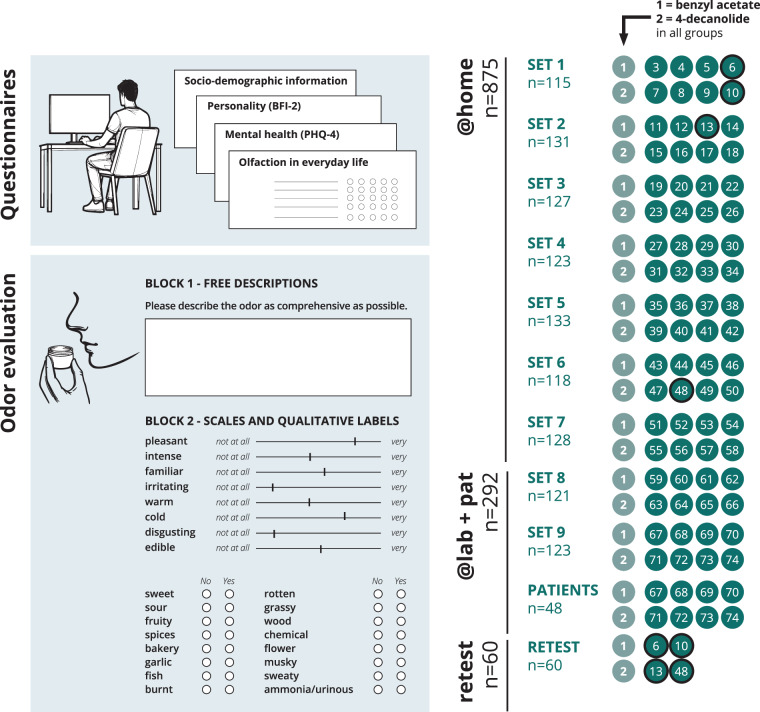
Overview of the study design and procedures. The study has been conducted in Germany and in the German language only, instructions and scales are presented in English here for better readability. First, all participants filled in questionnaires using an online survey implemented via LimeSurvey. Then, participants were assigned to one out of nine odor sets, containing a subset of ten out of the total 74 mono-molecular odors. The odor samples were provided in small plastic containers. Two “anchor odors” were used in all odor sets for comparison between groups, the other eight odors differed between groups. The participants first gave a free description for each odor (in German) and then evaluated them again using eight visual analogue scales and 16 further qualitative labels obtained from Keller & Vosshall^10^. The labels from Keller & Vosshall were translated into German for the study, but are displayed in English in this figure. 875 participants for odor sets 1 to 7 (@home) performed the questionnaires and odor evaluation at home using a prepared test kit. 244 healthy participants and 48 patients for odor sets 8 and 9 (@lab) performed the odor evaluation on site at the laboratory facilities. Another 60 healthy participants were recruited for a retest study: Each smelled at a selection of six odors chosen based on interim results of data collection from the nine odor sets on two measurement occasions separated by approximately one week.

With this dataset, we aim to provide a comprehensive database of olfactory perception based on a large-scale approach. This study involved the participation of 1,227 individuals who evaluated 74 mono-molecular odors by free description and rating scales. Additionally, the participants completed questionnaires regarding their socio-demographic background, personality, and the importance of olfaction in their everyday lives (see schematic Fig. 1). The odorants were chosen to span on a broad range of chemical composition. Furthermore, we checked for an overlap of our chosen molecules and the perceptual dimensions investigated with previous datasets such as the one from Keller & Vosshall^10^ to enable pooling and enlarging the current database of olfactory perception. With the dataset described here, we thus expect to provide a valuable tool for further investigations on the relation of the “structure-percept problem” as well as a useful database for olfactory research.

## Methods

### Study design and sample

#### Study design and rationale

**Fig. 2:**
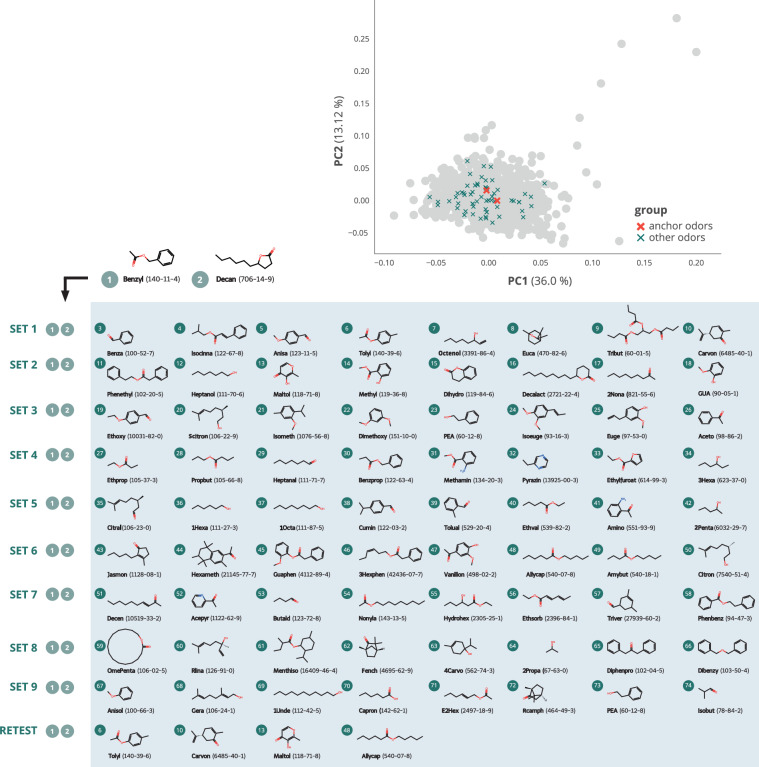
Odors selected from the chemical space. Odors were chosen from a list of commonly used odors in industry and research provided in Khan *et al*.^4^ and used for conducting a principal component analysis on a range of physical and chemical properties as described in Bierling *et al*.^26^. The position of the molecules chosen for this study in the physico-chemical odor space is indicated as “x”. The red “x” show the two anchor odors used across all groups. Two-dimensional illustrations of the molecules are shown for each group. Molecule names are given as abbreviative forms, see Data Record 2, together with the corresponding CAS number in brackets. Note: The odor tributyrin (set 1, odor number 9) has later been replaced by 2-phenyl ethanol (PEA) due to chemical reactivity with the plastic containers. For the same reason, PEA was used in odor set 3 (odor number 23) instead of the originally chosen odor cuminol (4Isoprop). For details see section “Odor selection”.

The aim of this study was to create a database containing the odor perception of as many laypersons as possible for as many mono-molecular odorants as possible. To achieve this goal, we followed a nested design by collecting odor ratings from 1,080 healthy individuals, with each odorant being rated by at least 120 individuals, thus, resulting in nine sampling groups. Each sampling group received an odor set consisting of 10 mono-molecular odorants: eight unique odorants and two “anchor odorants”, which were used in all odor sets. Thus, a total of 74 odorants has been investigated in this study (9 odor sets  × 8 unique odorants  + 2 anchor odorants  = 74 odorants). The study was primarily run at the participants’ homes (“@home” group). Odor sets 1–7 (recruitment goal n = 840) were provided to the participants as test kits containing 10 odorant samples in plastic containers and a link to an online survey, which enabled the participants to conduct the study at home. To ensure the validity of this approach, we aimed to invite 240 participants for the odor sets 8 and 9 (“@lab” group) to our laboratory facilities. This enabled us to compare anchor odor ratings between “@home” and “@lab”. For testing reliability, we invited 60 additional participants (“retest” group), who performed the study with a subset of odorants at two appointments separated by approximately one week. This design enables analyses of olfactory perception within-subject, between-subject, and temporal within-subject stability. The 72 unique odorants were randomly assigned to the nine odor sets with one exception: Some odorants were identified as being too dangerous (e.g., irritating for the skin if touched) for an uncomplicated testing at the homes of the participants without supervision. These odorants were assigned to the odor sets 8 and 9, which were tested under supervision at our laboratories. For the retest sample, a selection of six odorants out of the 74 was made based on interim results of the main study (details given below). Finally, in addition to the 1,080 healthy individuals, we aimed to recruit 120 patients with reduced olfactory performance (patient group) to compare healthy and pathologically altered olfactory perception. The patients received odor set 9 to enable direct comparisons between healthy participants and patients. An overview of the study design with the sampling groups and assigned odor sets is given in Figs. 1 and 2.

#### Inclusion and exclusion criteria

We included healthy individuals within an age range of 16 to 45 years who were proficient in German and reported normal olfactory ability, as well as patients with a reduced olfactory performance. Olfactory ability was additionally tested with the Sniffin Sticks®(Burghart Messtechnik, Holm, Germany) test battery in all @lab participants and in all patients. Patients were excluded if they fell outside of the range for hyposmia, i.e., values below 16 or above 30. Further exclusion criteria were pregnancy as well as - for the healthy participants - significant health impairments associated with interference with olfactory function (e.g., diabetes mellitus, renal insufficiency); acute or pronounced chronic inflammation of the nose (including acute COVID-19 infection) and paranasal sinuses; other relevant pre-existing or concomitant diseases in the ear, nose and throat area or underlying brain-organic diseases.

#### Participants and recruitment

In total, we recruited 1,227 participants, thus, over-achieving our recruitment goal of 1,080 healthy individuals. The minimum group occupancy rate for healthy participants was 95.8% (Fig. 1). Participants were recruited through a study website, via public notices, posts in forums and social networks, announcements in lectures at the universities of Dresden and Jena, and notices in the outpatient clinic of the Interdisciplinary Center for Smell and Taste. Participants received moderate financial compensation for participation (@lab, retest), or a little present (@home; sticker, keychain, and possibility to participate in a lottery to win vouchers with a value of 50€). Patients received no financial compensation. For each of the nine odor sets, we stopped the recruitment as soon as n = 120 participants had finished taking part in the whole study. However, as on average one third of the recruited @home participants dropped out, we handed out more @home test kits than necessary to compensate dropouts. Similarly, for the same reason we arranged more @lab appointments than necessary to compensate “no show” participants. This resulted in a slight over-recruitment for some odor sets (+35 for @home and +4 for @lab). On the other hand, although the @home participants were informed beforehand about inclusion and exclusion criteria (see section below), some of the participants still named exclusion criteria after receiving the test kit when conducting the study at home. Thus, for some odor sets, fewer than 120 participants fulfilled the inclusion-/exclusion criteria. 87 participants across all @home participants reported one or several exclusion criteria: 76 reported a current or chronic infection of the nose or sinuses, five a general health impairment related to olfaction, three an ENT (ear-nose-throat) related health impairment and three were pregnant. The data for the 87 excluded participants are also accessible via the provided files, but not counted or reported here in the validation section. For the @lab participants, inclusion and exclusion criteria were double-checked before invitation to the on site appointment and checked again on arrival. Thus, no participants were excluded for @lab. In the end, we included 875 participants (+87 excluded) for @home and 244 participants for @lab. Concerning the patient sample, recruitment was more challenging. In order to enable better comparability with the healthy group, the age range was also set at 16-45 years. However, patients presenting themselves in the clinic with olfactory disorders are typically older. As the recruitment for this study was during different phases of the ongoing COVID-19 pandemic (2021–2023), fluctuating access to young, hyposmic patients was given. Further complicating recruitment, not all patients in the respective age were eligible. Anosmic individuals, i.e., patients who were diagnosed having none or a very reduced sense of smell^19^, were not included for ethical reasons. For them, the task of giving proper descriptions and ratings of odors, which they cannot perceive at all is very frustrating and discouraging. The final sample size for this group was n=48 patients.

### Procedure

#### Ethical approval and informed consent

This study has been approved by the respective Ethics Committees of the TU Dresden (process number 361082020) and the Friedrich-Schiller-Universität Jena (process number FSV 23/019). At the beginning, the subjects were informed online about the aim and procedures of the study as well as the data protection measures taken. For all participants younger than 18, written informed consent from the parents had been obtained beforehand in addition to the informed consent given by the under-aged participants themselves. Participants have been explicitly informed and asked for consent of publishing the dataset in an open access publication. To protect the participants’ anonymity, we checked the sociodemographic variables (age, gender, nationality, mother tongue) for rare combinations, which might identify individuals. Moreover, the free descriptions of odors were screened for potentially identifying information during the standardization process described below.

#### Online survey

All recruited @home and @lab participants were given access to an online survey implemented via the TU Dresden hosted LimeSurvey platform before the actual experiment. All questionnaires and odor evaluations have been conducted in German. Participants were first asked to complete a short questionnaire to record demographic data (age, gender, nationality, native language, education level, professional level, smoking habits, and subjective smelling ability at the testing day on a scale from 0 = very low to 100 = very good olfactory ability). In addition, a screening of mental health was performed using the Patient Health Questionnaire 4^20^ and the presence of a (recovered) COVID-19 infection or any exclusion criteria was asked. Subsequently, several other questionnaires were completed online: The participants’ personality was assessed with the long 60-item form of the Big Five Inventory-2^21^, the role of olfaction in the everyday lives of the participants was evaluated from a questionnaire on olfactory dreams^22,23^ and a questionnaire on the individual odor significance^18^. After completion of the online questionnaires, the olfactory evaluation took place either at the participants’ home (@home) or in our laboratory facilities (@lab). All obtained data (socio-demographic data, questionnaires) are described in Data Record 1.

#### Odor evaluation

The @home participants received a set of ten labeled plastic containers (diameter of 3cm) loaded with 0.5ml of the different odors on a gauze swab and a detailed instruction including a link to an online survey. The participants were instructed to conduct the odor evaluation in an undisturbed place, which is as free as possible from odors in the surroundings. They were further instructed not to smoke, eat or drink coffee for at least 30 minutes before conducting the experiment. To ensure variability in the order of presentation of odors, we created four randomization groups (A, B, C, D) for each of the nine odor sets. Hence benzyl acetate, for example, could appear as the first odor in one randomization group and the fifth in another. Participants received the 10 labeled containers and were instructed to evaluate them in chronological order. To resolve the order of appearance of the odors later, the participants’ randomization group was included in their participant code. The odor evaluation was implemented via an online survey (LimeSurvey) and the instructions given in the survey also structured the evaluation procedure. In a first block, the participants were instructed to describe the smell of the odors freely. For this purpose, the participants were asked to open the labeled plastic container with the respective number from 1 to 10, smell it for a few seconds, and describe the odor of the sample as precisely as possible in a free-text field before closing the container and opening the next one. In the second block, the participants were asked to smell each odor again and rate it on eight visual analogue scales (*pleasantness, familiarity, intensity, irritability, edibility, disgust, warmness, coldness*; German scales used: *angenehm, bekannt, intensiv, reizend, essbar, Ekel erregend, warm, kalt*) and 16 qualitative descriptors, before closing the container and opening the next one. For the analogue scales, the slider was set exactly in the middle between the anchors “not at all” to “very”, but had to be clicked once, if the middle position was to be targeted. For the sixteen qualitative descriptors, participants were asked to mark “yes” or “no” (*sweet, sour, fruity, spices, bakery, garlic, fish, burnt, decayed, grass, wood, chemical, flower, musky, sweaty, ammonia/urinous*; German scales used: *süß, sauer, fruchtig, würzig, nach Backware, nach Knoblauch, nach Fisch, verbrannt, verwest, nach Gras, nach Holz, chemisch, blumig, moschusartig, nach Ammoniak/Urin*). The choice of the visual analogue scales was made in alignment with previous studies in the field^24,25^ and represents a mixture of very commonly used scales to characterize olfactory quality (*pleasantness, intensity, familiarity, edibility, disgust*) and trigeminal quality (*warm, cold, irritating*). The choice of qualitative labels was made in alignment with the qualitative descriptors used in Keller & Vosshall^10^, however, to reduce the strain of the participants, labels were asked binary (yes/no) instead of using visual analogue scales as in Keller & Vosshall^10^. The @lab participants and patients performed the odor evaluations in the laboratory facilities of the University Hospital in Dresden. Participants were instructed by the investigator on site and then performed exactly the same procedure as the @home participants with the only difference, that the investigator remained passively in the room (e.g., to answer questions if participants had any) and that a test of olfactory ability was conducted after the odor evaluation (described in next section). The retest participants conducted the same procedure as the @lab participants and the patients, but without the test of olfactory ability and on a subset of six chosen odors, on two consecutive appointments separated by approximately a week. All obtained data (odor evaluations main study and retest) are described in Data Record 1.

#### Test of olfactory ability

The Sniffin’ Sticks test is a standard procedure that uses felt-tip pens loaded with odors, each of which is briefly opened and presented to the subject for smelling^19^. The total sum score is made up of three subtests for odor threshold, discrimination, and identification. In each subtest, a maximum of 16 points can be achieved, i.e. the total scale ranges from 0 to 48 points. A sum score of at least 31 points indicates normal olfactory functioning (normosmia), values between 16 and 30 refer to a reduced olfactory functioning (hyposmia) and a score below 16 refers to a very reduced or absent olfactory functioning (anosmia).

### Odor molecule selection

In total, 74 mono-molecular odors were chosen, which differ in their chemical composition and overlap with other studies conducted on olfactory perception (see Fig. 2). In order to achieve a list of chemically diverse molecules, we used the physico-chemical “odor space” described in Bierling *et al*.^26^, which was based on chemical and physical descriptors for 1389 odor molecules obtained from Khan *et al*.^4^. For the choice of molecules in this study, we first calculated an average distance vector between the 1389 odor molecules by calculating the distance of each molecule to all other molecules within the three-dimensional space derived from the first three principal components of the odor space^4,26^. The resulting list of molecules was then sorted by the distance vector, i.e., from the highest averaged distance of a molecule to all other molecules in the odor space to the lowest averaged distance to all other molecules. From this list, we successively chose odors (starting from the molecule with the highest average distance vector), which showed an overlap with the molecules studied in Bierling *et al*.^26^, Khan, *et al*.^4^, Keller and Vosshall^10^ and Lötsch, *et al*.^24^ in order to enable as many direct comparisons of olfactory perception with previous studies as possible. As a last step, odors were checked for their availability, price and safety. As odors for odor sets 1 to 7 were intended for testing at the participants’ homes, we omitted odors, which were in any way hazardous, i.e., corrosive.

For two specific odor molecules, tributyrin and cuminol, a chemical reaction with the plastic container was observed after some time of storage. This led to some participants being unable to open the plastic containers and the danger of liquid dripping from the containers. In case of tributyrin, 83 of the 115 participants still gave descriptions on the odor and these can be found in the dataset, whereas for cuminol, the problem already became apparent after a few days of storage during the preparation of the test kits, thus, the odor was exchanged before any examinations took place. Both tributyrin and cuminol were replaced by 2-phenylethanol, which is an often used odor in olfactory research and is also used in groups 9 and 10.

Benzyl acetate and 4-decanolide were chosen as “anchor” odors, which remained the same for all participants. Those molecules were selected, because both have been shown to work in experiments with a large variety of participants, including participants with reduced olfactory abilities^24^.

For the retest study, we chose six odors based on their perception measures pleasantness, familiarity, irritability and disgust after approximately 80% of the data collection for @home and @lab testings had been finished. Based on the interim results, we chose to include the (at that time) most and least pleasant, familiar, irritating and disgusting odors, which resulted in the four odors maltol (most pleasant, least irritating), (R)-(-)-carvone (most familiar, least disgusting), allyl caproate (second-most disgusting, least familiar) and p-tolyl acetate (least pleasant, most irritating and most disgusting). Furthermore, we included the two anchor odors 4-decanolide and benzyl acetate, which ranked medium for the four perceptual measures with a high variability. The final odor selection, as well as further information on the used substances is made available (see Data Record 2).

#### Concentration of the odors

All 74 odors were piloted to be similar in their intensity and were then used in the obtained ideal concentrations (e.g., lower concentration for very intense odors, higher concentration for more subtle odors). First, the two anchor odors benzyl acetate and 4-decanolide were piloted to have an average intensity of around 50 on a scale from 1 = not perceivable to 100 = extremely intense in n=18 piloting participants. Afterwards, all other odors were piloted group-wise with at least n = 10 participants per group to match the intensity of benzyl acetate and 4-decanolide. As a starting concentration for piloting, if available, the suggestion provided by the database of The Good Scent Company was used as a first estimate of ideal intensity. If none was available, a moderate intensity was first agreed upon between the two study investigators A.L.B. and M.R. If the piloting showed that an odor was very intense or very subtle compared to the anchor odors, the odor was used in a more diluted or more concentrated form in the following steps “undiluted”, “1:1”, “1:10”, “1:100” to “1:1000”. Results from intensity piloting are provided in Data record 3.

## Data Records

The dataset is available at zenodo (14727277)^27^. In total, the record comprises six files: One main data file (**data.xslx**) containing the data (i.e., socio-demographic data, questionnaires, odor evaluations) from the main study and the retest study, another file containing information on the odor stimuli (**odors.xlsx**), a file for the intensity piloting results (**intensity_piloting.xlsx**), a file containing a detailed list of all variables (**variables-dictionary.xlsx**), a file containing the most frequent free descriptions with an English translation (**free_descriptions_translated.xlsx**) and the jupyter notebook file (**analyses.ipynb**), which contains the analyses and code for creating the illustrations shown in this manuscript.

### Data record 1 - data.xlsx

The dataset is provided in an excel spreadsheet (XLSX) and is structured in long format, i.e., each row represents one participant’s ratings of one odor (for retest: at one measurement occasion), with a total of ten rows per participants for the main study (10 odors  × 1 measurement) and twelve rows per participant in the retest study (6 odors  × 2 measurement occasions). The dataset is structured in four types of data, which are explained in detail in the variables dictionary: 1) identifiers of participants, odors and study groups, 2) socio-demographic variables, 3) results of the odor evaluation and 4) the questionnaire results as raw items and as aggregated scores.

As **identifiers** of the participants, the study (main study/retest), dataset type (home/lab/pat/retest), a code for each participant and the belonging to the different odor groups and randomization groups are given. For the stimuli, the molecule and order of appearance of the molecules are given, as well as the time of storage of the @home odors in the main study and the time between test and retest appointment for the retest study.

For the **socio-demographic background**, we asked for the participants’ age, gender, highest school and professional education, as well as smoking habits, and if participants ever suffered from a COVID-19 infection. Nationality and mother tongue were also asked, however, as only a very small minority of participants did not grow up in Germany (3.3%) or speaking German as a native language (2.3%), these two variables have been omitted from the dataset to ensure anonymity of the participants.

For the **odor evaluation**, each participant was asked to give a free description for each odor. The free descriptions are provided in a standardized way for better comparability. Standardization was achieved by the following procedure. For each free description entry, all relevant odor description terms were identified. For example, for an entry “This odor smells like lemon, a little fruity, sour and remembers me of a cleaning agent.” the following terms were identified: “lemon”, “fruity”, “sour” and “cleaning agent”. Furthermore, for standardization, the terms were transferred to their so-called “lemmas”, i.e., the dictionary form of words sharing the same word stem and same meaning. For example, “sweetish” and “sweet” would both be coded as “sweet”. If a word is both used as an adjective and noun (e.g., “lemon” and “lemony”), the more frequent term was used. Negations were coded as “NEG-” for both lexical (“not”) and morphological (“un-”) negations. Modifiers indicating intensity, location, temporality or similar (e.g. “a little”, “very”, “at home”) were not included. For complex descriptions, which would change their meaning if split in several terms (e.g. “old people perfume”) were kept as one term. Specific brand names (e.g. “Colgate toothpaste”, “Mucosolvan cough syrup”) were also coded, but the more general term (“toothpaste”, “cough syrup”) without brand name was coded as an additional term to facilitate comparability between descriptions. Finally, all free standardized descriptions were checked again for consistency and redundancies again before storage in this data file. The raw unprocessed data for the free descriptions are not available in this dataset in order to ensure that no person identifying information is published, but can be obtained upon request from the authors. A file containing English translations of the most frequent German descriptions is given in a separate file (see Data Record 4).

Furthermore, the ratings of the odors on eight visual analogue scales (pleasant, intense, familiar, warm, cold, edible, disgusting, irritating) as well as on 16 qualitative labels (sweet, sour, fruit, spices, bakery, garlic, fish, burnt, decayed, grass, wood, chemical, flower, musky, sweaty, ammonia/urinous) obtained from Keller & Vosshall are presented. In addition, we also present the results of the Sniffin Sticks test batteries (threshold, discrimination, identification, total score) as well as the subjective olfactory performance.

Finally, the responses of the **questionnaires** on mental health, personality, individual odor significance, (self-)smelling behavior and olfactory dreams are provided both as the raw items and in an aggregated form. Details on the coding of answers are given in the variables dictionary.

### Data record 2 - odors.xlsx

A list of all substances used as odor stimuli is given in an excel spreadsheet (“odors.xlsx”) and provides one row per odor used in one group, together with further information on concentration, identifiers and catalogue numbers. The abbreviated code of the molecules is given for matching with the perception dataset.

### Data record 3 - intensity_piloting.xlsx

The data of the intensity piloting is provided in another excel spreadsheet (“intensity_piloting.xlsx”) in the long format, i.e., each row indicates one participant rating one odor substance’s intensity from 1 = not perceivable to 100 = very intensive. The starting concentration (odor:solvent) and volume used in the piloting is given, as well as the abbreviative code of the molecules for matching with the perception data.

### Data record 4 - free_descriptions_translated.xlsx

This dataset (“free_descriptions_translated.xlsx”) contains the top-ranked free descriptions together with an English translation. To provide this file, the German free descriptions from the main dataset (see Data Record 1) were grouped and sorted by frequency of naming across all odor sets and participants. From that, we identified the descriptions ranked 1 to 100, including ties, and translated them to English using back-and-forth translation. Discrepancies between translation and back-translation were resolved by discussion. Within this dataset, one row represents one description (column “free_description”) given by one participant (“code”) for one molecule (“molcode”) as well as the English translation (“translation”). There can be several rows per participant and molecule as participants typically used several descriptors in their free descriptions. Using these top 100 ranked descriptions, the translated dataset covers 26,115 out of the original 37,041 free descriptions, which represents 70.5% of all free descriptions.

### Sample composition

In total, the dataset comprises data from n = 1,227 individuals, who participated in the different study groups as outlined above. The sample is composed of young healthy individuals and patients with a reduced olfactory function. Across all study groups, the mean age was 26.18 years (SD = 7.61) with a gender distribution of 68.27% females, 31.21% males and 0.52% who identified other. The education level of the participants was generally high, with 81.11% having completed the highest secondary education track in Germany; 16.71% holding a degree from a German middle school, and 1.50% having another or (until that time) no completed educational degree. With regard to professional education, the majority of 45.23% of participants had no completed professional education, which is probably attributed to a high percentage of undergraduate university students in the study. A total of 34.79% of participants held university degrees, with 12.62% having obtained a Bachelor’s degree, 19.90% a Master’s degree and 2.27% a doctoral degree. Additionally, 18.00% had completed a vocational training diploma, and 2.00% were master craftsmen. Across all study groups, the majority of participants were non-smokers (80.12%), followed by occasional smokers (“sometimes”, 12.00%), and regular or frequent smokers (“several times a week”, 0.82%, “once a day”, 1.18%, “several times a day”, 5.90%). The prevalence of a (recovered) COVID-19 infection varied largely between groups. The recruitment for this study started with the groups 1, 2 and 8 in autumn 2021, when only a small percentage of the population had suffered from a COVID-19 infection. Accordingly, in those groups only around 20% of the participants had a COVID-19 infection in the past (see Table 1), whereas this percentage continuously increased with the recruitment period until late summer and autumn 2023, where groups 6, 7 and the retest group showed prevalences of around 70–80%. A detailed overview of the named socio-demographic variables within each study group is given in Table 1.

**Table 1:** Overview of socio-demographic background.

sampling group	@home							@lab		patients	retest
odor set	1	2	3	4	5	6	7	8	9	9	retest
**Age**											
*Mean*	26.99	26.77	23.99	24.78	25.38	25.99	22.95	27.74	28.88	33.95	27.17
*Std*	6.73	7.59	7.5	7.77	8.02	7.85	4.53	7.42	7.16	7.34	5.32
**Gender (%)**											
*Female*	71	86	92	88	87	82	103	60	83	29	41
	62%	66%	72%	72%	65%	69%	80%	50%	67%	60%	68%
*Male*	43	44	35	35	45	34	24	50	33	14	18
	37%	34%	28%	28%	34%	29%	19%	41%	27%	29%	30%
*Other*	1	2	1	1	1	1					1
	1%	2%	1%	1%	1%	2%					2%
**Highest education (%)**											
*none*	1	2	1	1	3						
	1%	2%	1%	1%	3%						
*lower secondary*	1	1	2	1	1	2					
	1%	1%	2%	1%	1%	2%					
*middle school*	6	26	27	36	37	9	2	20	21	3	3
	5%	20%	21%	29%	28%	8%	2%	17%	17%	5%	5%
*higher secondary*	104	104	96	86	92	108	123	87	93	57	57
	90%	79%	76%	70%	69%	92%	96%	72%	76%	95%	95%
*other*	1	1	1	2	1	1	1				
	1%	1%	1%	2%	1%	1%	1%				
**Highest professional education (%)**											
*none*	39	38	76	70	67	60	86	30	32	18	18
	34%	29%	60%	57%	50%	51%	67%	25%	26%	30%	30%
*vocational training*	11	26	15	22	24	15	18	35	32	4	4
	10%	20%	12%	18%	18%	13%	14%	29%	26%	7%	7%
*master craftsman*	5	1	2	1	3	1	7	2	1		1
	4%	1%	2%	1%	3%	1%	6%	2%	2%		2%
*bachelor*	23	18	13	8	11	18	11	19	18	16	16
	20%	14%	10%	7%	8%	15%	9%	16%	15%	27%	27%
*master*	35	41	19	19	28	20	11	18	28	19	19
	30%	31%	15%	15%	21%	17%	9%	15%	23%	32%	32%
*doctorate*	4	3	3	3	3	3	1	1	4	2	2
	3%	2%	2%	2%	2%	3%	1%	1%	3%	3%	3%
**Smoking behavior (%)**											
*non-smoker*	99	100	103	96	115	99	100	83	87	50	50
	86%	76%	81%	78%	86%	84%	78%	69%	71%	83%	83%
*sometimes*	11	14	15	15	10	12	21	17	17	7	7
	10%	11%	12%	12%	8%	10%	16%	14%	14%	12%	12%
*several times a week*	1	1	2	1	5	3	3				3
	1%	1%	2%	1%	4%	2%	5%				5%
*once a day*	2	1	2	3	1						
	2%	1%	2%	2%	1%						
*several times a day*	3	12	7	8	7	6	3	10	9		
	3%	9%	6%	7%	5%	5%	2%	8%	7%		
**COVID-19 infection (%)**											
*Yes*	22	29	72	77	81	98	104	25	69	35	47
	19%	22%	57%	63%	61%	83%	81%	21%	56%	73%	78%
*No*	93	102	55	46	52	20	24	85	47	8	13
	81%	78%	43%	37%	39%	17%	19%	70%	38%	17%	22%

Mean age and distribution of gender, highest education, highest professional education, smoking behavior and the prevalence of a (recovered) COVID-19 infection are given as frequencies and in percentage split by group.

## Technical Validation

In order to validate this dataset, we investigated the validity, objectivity and reliability of the odor evaluations. To this end, we compared our dataset to the results of another laymen perception dataset (Keller & Vosshall, 2016^10^). Furthermore, we checked for systematic differences of sampling group or condition using the anchor ratings. Finally, we tested the temporal stability of olfactory perception by correlating the retest study’s perception measures between the two measurement occasions. More details on each validation check are outlined in the following sections.

### Validity: Comparison with another laymen dataset

The file containing the laymen perception data of n = 55 participants of Keller & Vosshall can be downloaded via^10^. In total, n = 56 odors overlapped between the studies. As Keller & Vosshall investigated different concentrations of the odors, the highest available concentration was used for comparisons, as this was closest to our concentrations in this study. We calculated the percentage of our participants who rated “yes” for 19 qualitative perception measures, which were available in both studies: chemical, sweet, cold, warm, spices, fruit, edible, flower, sour, wood, musky, decayed, ammonia/urinuos, grass, bakery, sweaty, burnt, garlic and fish. For edible, warm and cold, we transformed our data to values between 0 and 1. For the data from Keller & Vosshall, the data were also transformed into values between 0 and 1. In order to evaluate differences between the two datasets, we the percentage of “yes” per measure and per molecule between the participants in Keller & Vosshall and our dataset. Overall, very similar patterns of ratings emerged, the results for each odor are visualized in Fig. 4. Furthermore, in order to evaluate whether participants from both datasets showed similar rank values for the 56 molecules, we calculated Spearman correlation coefficients for the associations between the visual analogue scale ratings pleasantness, intensity, edibility, warmth, coldness and familiarity which were present in both studies. As a result, we found significant positive associations for pleasantness (r = 0.68, p < 0.001), familiarity (r = 0.58, p < 0.001), edibility (r = 0.37, p < 0.01) and intensity (r = 0.28, p < 0.05), but none for warmth (r = −0.07, p = 0.59) and coldness (r = −0.20, p = 0.11).

**Fig. 3:**
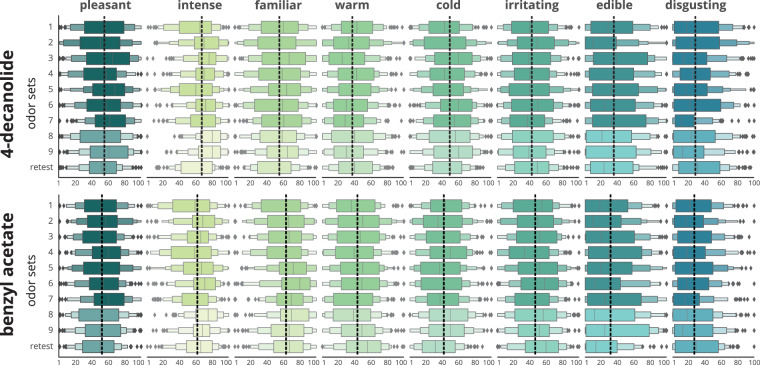
Comparison of the eight perception measures between the participants for the nine odor sets plus the retest group for the two anchor odors 4-decanolide and benzyl acetate. The black dotted lines indicate the average mean across all groups.

**Fig. 4:**
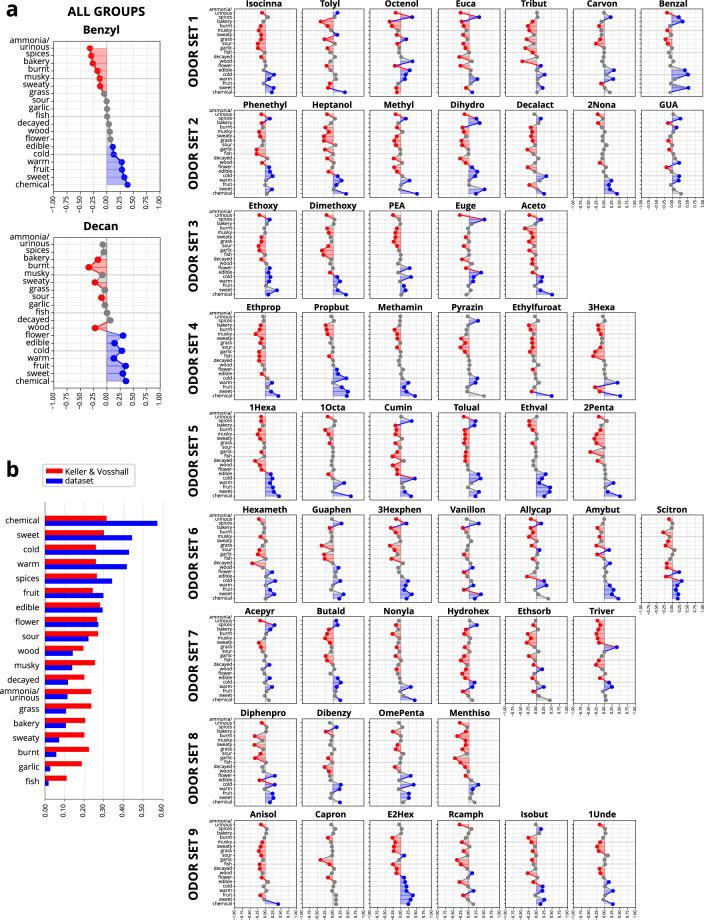
Comparison of qualitative descriptor usage with Keller & Vosshall (2016). a. For each molecule (abbreviations see Table 1), the proportion in percent of using each of the 19 qualitative labels is compared between the dataset presented here as well as the dataset by Keller & Vosshall^10^. In total, 55 odors were suitable for comparison. The two anchor odors benzyl acetate (Benzyl) and 4-decanolide (Decan) are highlighted in the left panel. Red coloured areas indicate a higher descriptor usage by participants in the dataset of Keller & Vosshall, blue colored areas indicate higher percentage of usage in this dataset. Grey points and grey shaded areas indicate a difference smaller than 10%. b. Overall frequencies of using each descriptor in both datasets, averaged across all molecules, which overlap between the two datasets. Red indicates frequencies in the Keller dataset, blue in this dataset. The order of descriptors is sorted by frequency (from top to bottom) of descriptor usage in this dataset.

### Objectivity: Context stability

When comparing the perception measures for the two anchor odors between the nine odor sets in all healthy participants, significant differences in the ratings were seen between groups for both anchor odors (see Table 2 and Fig. 3). For 4-decanolide, significant differences in the pleasantness, intensity, warmth, coldness, edibility and disgust ratings were found, with overall small effect sizes (all partial *η*^2^ < 0.06), except for a moderate effect for intensity (partial *η*^2^ = 0.063). For benzyl acetate, significant differences in the ratings were found for intensity, familiarity, irritability and edibility, with small effect sizes for all (all partial *η*^2^ < 0.06), however, intensity showing the highest effect size again (partial *η*^2^ = 0.048).

**Table 2:** Results from robust Welch one-way ANOVA testing the influence of odor set on each of the eight perception measures separately for the two anchor odors 4-decanolide and benzyl acetate.

dimension	4-decanolide					benzyl acetate				
	df1	df2	F	p-unc	np2	ddof1	ddof2	F	p-unc	np2
*pleasant*	8	446.7	**3.390**	0.001***	**0.024**	8	447.3	1.829	0.070	0.013
*intensive*	8	447.3	**8.603**	0.000***	**0.063**	8	447.3	**6.509**	0.000***	**0.048**
*familiar*	8	447.1	1.621	0.116	0.012	8	447.0	**2.966**	0.003**	**0.022**
*warm*	8	447.0	**2.510**	0.011*	**0.017**	8	447.7	1.507	0.152	0.011
*cold*	8	447.1	**3.348**	0.001***	**0.023**	8	447.6	1.710	0.094	0.013
*irritating*	8	447.1	1.402	0.193	0.010	8	447.3	**2.458**	0.013*	**0.018**
*edible*	8	447.1	**4.034**	0.000***	**0.027**	8	447.2	**2.280**	0.021*	**0.016**
*disgusting*	8	446.1	**3.535**	0.001***	**0.021**	8	447.1	1.088	0.370	0.007

df = degrees of freedom, p-unc = uncorrected p-value, np2 = partial *η*^2^

Significance marked according to *p < 0.05, **p < 0.01, ***p < 0.001.

Games-Howell post hoc comparisons showed significant differences in the perception of the two anchor odors 4-decanolide and benzyl acetate between the nine odor sets; mostly with low effect sizes of Hedges’ g < 0.5 (see Table 3). For both anchor odors, intensity showed the most and largest differences between the odor sets, with moderate effect sizes for a few comparisons. For all significant comparisons refer to Table 3).

**Table 3:** Results from Games-Howell post-hoc comparisons for the two anchor odors 4-decanolide and benzyl acetate.

dimension	4-decanolide									
	A	B	mean(A)	mean(B)	diff	se	T	df	p-corr	hedges
*pleasant*	2	5	49.59	61.79	−12.21	3.61	−3.39	246.39	0.023*	−0.42
	2	7	49.59	61.79	−12.20	3.57	−3.42	241.03	0.021*	−0.43
*intensse*	1	3	59.26	72.29	−13.03	3.04	−4.29	217.33	<0.001***	−0.56
	1	6	59.26	69.73	−10.47	3.08	−3.41	216.08	0.022*	−0.45
	1	8	59.26	76.37	−17.11	2.97	−5.75	207.18	<0.001***	−0.76
	1	9	59.26	74.74	−15.48	3.03	−5.10	214.01	<0.001***	−0.67
	2	5	68.80	59.05	9.74	3.03	3.22	254.63	0.038*	0.40
	3	5	72.29	59.05	13.23	2.88	4.59	246.21	<0.001***	0.57
	4	5	68.44	59.05	9.39	2.93	3.20	247.41	0.041*	0.40
	5	6	59.05	69.73	−10.68	2.92	−3.66	241.11	0.009**	−0.46
	5	8	59.05	76.37	−17.31	2.81	−6.16	236.16	<0.001***	−0.78
	5	9	59.05	74.74	−15.68	2.88	−5.45	241.42	<0.001***	−0.69
	7	8	66.01	76.37	−10.36	2.66	−3.90	234.90	0.004**	−0.50
	7	9	66.01	74.74	−8.73	2.73	−3.20	238.82	0.041*	−0.41
*warm*	1	3	43.65	32.56	11.09	3.34	3.32	233.96	0.029*	0.43
	1	7	43.65	33.52	10.12	3.19	3.18	231.95	0.044*	0.41
*cold*	1	3	43.43	55.17	−11.74	3.43	−3.42	233.91	0.02*	−0.44
	1	6	43.43	55.90	−12.47	3.36	−3.71	225.94	0.008**	−0.49
	4	6	45.03	55.90	−10.87	3.45	−3.15	235.00	0.047*	−0.41
*edible*	2	3	24.76	43.28	−18.52	4.05	−4.58	241.40	<0.001***	−0.58
	2	4	24.76	38.32	−13.56	3.92	−3.46	244.07	0.018*	−0.44
	2	5	24.76	40.06	−15.30	3.93	−3.90	252.33	0.004**	−0.48
	2	7	24.76	42.30	−17.54	4.08	−4.30	241.55	<0.001***	−0.54
	2	9	24.76	37.64	−12.88	4.03	−3.20	234.12	0.041*	−0.41
*disgust*	2	7	33.56	20.11	13.45	3.37	3.99	223.28	0.003**	0.50
	4	7	30.48	20.11	10.38	3.06	3.39	231.58	0.023*	0.43
	6	7	31.59	20.11	11.49	3.42	3.36	202.13	0.025*	0.44
	7	8	20.11	31.21	−11.11	3.36	−3.30	203.41	0.03*	−0.43
	**benzyl acetate**									
**dimension**	**A**	**B**	**mean(A)**	**mean(B)**	**diff**	**se**	**T**	**df**	**p-corr**	**hedges**
*intense*	1	2	54.425	66	−11.575	3.382	−3.422	214.378	0.021*	−0.446
	1	6	54.425	66.661	−12.236	3.465	−3.531	215.189	0.015*	−0.467
	1	8	54.425	71.36	−16.935	3.268	−5.182	197.787	<0.001***	−0.687
	1	9	54.425	65.256	−10.832	3.329	−3.253	205.11	0.035*	−0.43
	2	4	66	53.639	12.361	3.377	3.661	227.864	0.009**	0.465
	3	8	61.153	71.36	−10.206	2.803	−3.641	233.814	0.01**	−0.467
	4	6	53.639	66.661	−13.022	3.46	−3.764	227.384	0.007**	−0.484
	4	8	53.639	71.36	−17.72	3.262	−5.433	211	<0.001***	−0.696
	4	9	53.639	65.256	−11.617	3.323	−3.495	218.26	0.016*	−0.448
	7	8	59.556	71.36	−11.803	2.706	−4.361	235.728	<0.001***	−0.561
*familiar*	1	6	58.354	70.304	−11.95	3.73	−3.204	225.993	0.041*	−0.423
*irritating*	4	6	38.59	52.33	−13.74	3.633	−3.782	234.833	0.006**	−0.49

For the sake of readability, only significant comparisons are given. All results can be obtained from the analyses notebook provided.

A, B = odor sets for comparison, mean(A), mean(B) = mean value of this dimension for this odor set,

diff = difference between means, df = degrees of freedom, p-corr = corrected p-values, hedges = hedges effect size;

Significance marked according to *p < 0.05, **p < 0.01, ***p < 0.001.

**Fig. 5:**
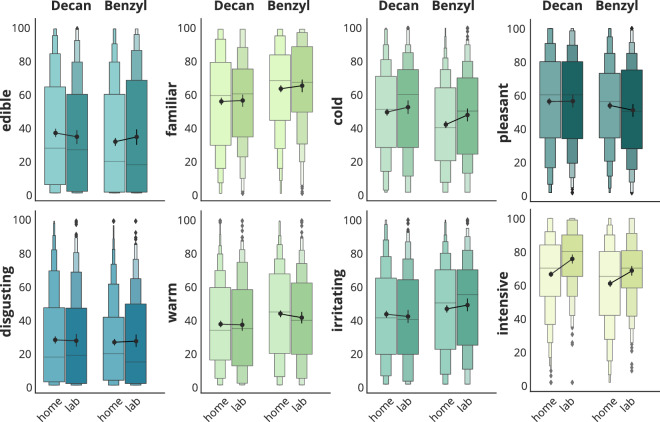
Comparison between the two conditions @home and @lab. Each plot illustrates the distribution of one of the eight perception dimensions’ ratings in boxenplots for the two anchor odors used across all groups for the two conditions @home and @lab. A lineplot connecting the mean values is given in each subplot.

Concerning the experimental setting, no significant difference in mean or variance were found for the perception measures pleasantness, warmness, irritability, edibility, and disgust between @home and @lab measurements. However, significant differences in the mean values were found for intensity (t = −6.77, p < 0.001, d = −0.37) and coldness (t = −3.01, p < 0.01, d = −0.16) with low effect sizes. Thus, participants in the @home setting rated odors as slightly less intense and less cold. Furthermore, a significant difference in the variance was found for intensity (t = 19.78, p < 0.001). See Fig. 5.

### Reliability: Temporal stability

**Fig. 6:**
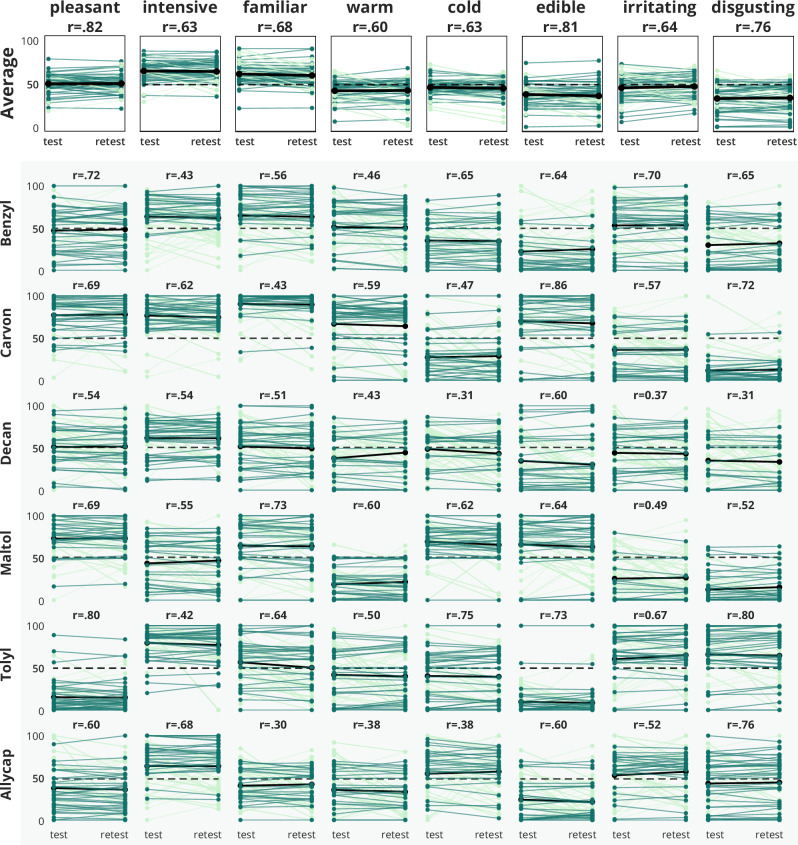
Temporal stability of odor perception. The top panel illustrates the average ratings of the eight visual analogue scales at test and retest appointment as connected lineplots. Each connected line indicates ratings from one participant. The dark green color indicates that no substantial change has occurred (absolute change <10%), light green lines indicate increases or decreases by at least 10%. The bold black lines indicate the average ratings across participants. Below the top panel, the ratings are shown for each odor, thus, in each plot one line represents the rating of this dimension for this odor by one participant at the two measurement occasions. On top of each plot, the correlation coefficient is given. A dotted line indicates a value of 50, which is exactly the middle position indicating a neutral opinion.

Retest reliability was estimated from n = 60 participants, who were asked to rate and describe six of the odors (benzyl acetate, 4-decanolide, maltol, allyl caproate, (R)-(-)-carvone and p-tolyl acetate) at two appointments within a time span of a week (M = 7.37 days, SD = 1.37). Spearman correlation coefficients showed significant associations of all perception measures with the retest measurement at the follow-up appointment (see Fig. 6 and Table 4) for all chosen odors. The highest correlation coefficients were found for pleasantness (overall correlation across odors between test and retest r = 0.81, p < 0.001), edibility (r = 0.79, p < 0.001) and disgust (r = 0.77, p < 0.001). The least stability was seen for warmness (r = 0.60, p < 0.001), coldness (r = 0.63, p < 0.001) and intensity (r = 0.63, p < 0.001) ratings. Furthermore, the two odors 4-decanolide and allyl caproate showed on average a less stable pattern of retest reliability compared to the other odors.

**Table 4:** Correlations (Spearman) of perception measures with follow-up appointment (n = 60).

Odor	pleasant	intensive	familiar	warm	cold	edible	irritating	disgusting	Mean
*benzyl acetate*	0.74***	0.45***	0.58***	0.44***	0.64***	0.64***	0.69***	0.67***	**0.61**
*4-decanolide*	0.51***	0.51***	0.48***	0.42***	0.31*	0.59***	0.39**	0.37**	**0.45**
*maltol*	0.69***	0.57***	0.73***	0.54***	0.58***	0.70***	0.54***	0.58***	**0.62**
*allyl caproate*	0.65***	0.68***	0.27*	0.38**	0.43***	0.48***	0.53***	0.76***	**0.53**
*(R)-(-)-carvone*	0.74***	0.58***	0.61***	0.55***	0.40**	0.83***	0.57***	0.78***	**0.63**
*p-tolyl acetate*	0.72***	0.46***	0.63***	0.49***	0.76***	0.56***	0.68***	0.81***	**0.64**
**Across odors**	0.81***	0.63***	0.70***	0.60***	0.63***	0.79***	0.65***	0.77***	

Significance marked according to *p < 0.05, **p < 0.01, ***p < 0.001.

## Usage Notes

The code needed to perform the analyses and illustrations shown in this manuscript is provided as a Jupyter Notebook (analyses.ipynb), operating with Python 3.7 and using the packages *pandas*, *numpy*, *matplotlib*, *statsmodels*, *scipy*, *pingouin*, *seaborn*, *PIL* and *rdkit*. For further adaptation of the illustrations in this manuscript, Adobe Illustrator 2024 and Adobe InDesign 2024 were used. In order to reproduce the analyses, download the jupyter notebook file (analyses.ipynb) and the three files data.xlsx, odors.xlsx and intensity_piloting.xlsx in the same directory. The files are loaded as dataframes from the original excel files in the notebook. For the comparisons with the dataset by Keller & Vosshall, the original dataset can be obtained via^10^.

## Data Availability

The code created to perform the analyses and creation of illustrations in this manuscript is available at^27^.
